# Editorial: The Challenge Posed by New Synthetic Opioids: Pharmacology and Toxicology

**DOI:** 10.3389/fphar.2019.00563

**Published:** 2019-05-21

**Authors:** Simona Pichini, Simona Zaami, Roberta Pacifici, Adriano Tagliabracci, Francesco Paolo Busardò

**Affiliations:** ^1^National Centre on Addiction and Doping, Istituto Superiore di Sanità, Rome, Italy; ^2^Section of Legal Medicine - SAIMLAL Department, Sapienza University of Rome, Rome, Italy; ^3^Section of Legal Medicine, Department of Excellence SBSP - University “Politecnica delle Marche”, Ancona, Italy

**Keywords:** new synthetic opioids, fentanyl, pharmacology, toxicology, analgesic opioids

Diverted prescription opioid analgesics (e.g., oxycodone, hydrocodone, hydromorphone), failed opioid drug candidates (e.g., benzamide derivatives), and various legal and illegal fentanyl analogs (e.g., acetyl fentanyl, furanylfentanyl, carfentanil) constitute the class of New Synthetic Opioids (NSOs), which is currently posing a global public health threat (Pichini et al., 2018).

Due to the low cost of materials and equipment required for clandestine laboratory production and enormous profit potential, NSOs are establishing a strong position on the illegal drug market as stand-alone products, adulterants in heroin, or constituents of counterfeit prescription medications. Recently, NSOs have been involved in a significant spike of acute intoxications (classic opioid toxidrome) and overdose deaths in North America, challenging healthcare professionals, law enforcement agencies fighting against their diffusion, and policymakers trying to restrain their use (Marchei et al., 2018; Busardò et al., 2019).

Since there is little information available regarding the pharmacology and the toxicology of NSOs in abuse settings, the main purpose of this Research Topic was to fill the current knowledge gap. The topic covers basic scientific, epidemiological, and clinical aspects of NSOs and includes 3 reviews, 3 mini-reviews, 1 original article, 2 case reports, and 1 opinion.

The Research Topic begins with the opinion of Pichini et al. on the health risks entailed in the emergence of illicit fentanyl mixes onto the European drug market, following the recent spike in overdose deaths in North America. To fight against this incoming threat, the authors advocated for the improvement of epidemiological surveillance and data sharing through National and International Early Warning systems and various communication platforms, and the publication of analytical methodologies for the identification of fentanyl analogs and metabolites in ante- and post-mortem cases.

Indeed, Schifano et al. demonstrated that the occurrences of fentanyl misuse, abuse, dependence, and withdrawal-related adverse drug reactions increased over time on international databases, with the most represented adverse reactions being “drug dependence,” “intentional product misuse,” and “drug abuse” with most cases involving adult males and the concomitant use of other prescribing/illicit drugs.

This latter occurrence was addressed by Pérez-Mañá et al. who reviewed drug-drug interactions with NSOs through pharmacokinetic and pharmacodynamic mechanisms, and discussed the role of naloxone, an opioid receptor antagonist, as an antidote to the NSO toxidrome. The authors recommended that medical doctors prescribing potentially abused opioids should be aware of the life-threatening risks induced by drug-drug interactions with NSOs to prevent new cases of intoxication.

With respect to fatalities caused by fentanyl and derivatives (e.g., acetyl fentanyl, butyryl fentanyl, carfentanil, furanyl fentanyl) and non-traditional opioid agonists (e.g., AH-7921, MT-45, U-47700), Concheiro et al. reviewed the current data available on the post-mortem toxicology of synthetic opioids and their chemical and pharmacological properties. The review includes pharmacokinetic parameters (metabolism), post-mortem redistribution, and stability studies in post-mortem samples.

Two single post-mortem cases were then reported. In the first case, Cannaert et al. described a novel *in vitro* opioid activity reporter assay based on μ-opioid receptor activity and a sensitive bioanalytical method for the determination of carfentanil in a fatal intoxication, reporting the highest carfentanil concentrations in a post-mortem case: 92 ng/mL in whole blood, 2.8 ng/mL in urine, and 23 ng/mL in vitreous humor. In the second case involving a man with previous history of drug addiction, Gerace et al. detected U-47700, a strong μ-opioid agonist with a 7.5-fold higher potency than that of morphine, in blood (380 ng/mL), urine (10,400 ng/mL), and pubic hair (5.7 ng/mg) using a new ultra-performance liquid chromatography tandem mass spectrometry method.

Wilde et al. reviewed the metabolic profiles and pharmacological potencies of new fentanyl analogs. Since only limited to no information on the metabolism of fentanyl analogs is available, the authors hypothesized and anticipated the metabolism of new compounds taking into consideration the well-characterized metabolism of pharmaceutically or illicitly used analogs, which generally involves phase I reactions such as hydrolysis, hydroxylation (and further oxidation steps), N- and O-dealkylation, and O-methylation and phase II metabolic reactions such as glucuronide or sulfate conjugation.

Solimini et al. reviewed the available information on the pharmacological properties of non-fentanyl NSOs, including U-47700, U-49900, AH-7921, and MT-45, providing a better understanding of these compounds, particularly on the toxicity and dangerous adverse effects in users.

Further with respect to non-fentanyl derived NSOs, Cardia et al. summarized the pre-clinical and clinical characteristics of hydrocodone. Pharmacokinetic aspects (terminal half-life, maximum serum concentration, and time to maximum serum concentration) and the influence of metabolic genetic polymorphism in analgesic response to the drug has been illustrated and discussed.

Finally, Gilardi et al. reported the pre-clinical and clinical findings on the implications of parental exposure to NSOs for their offspring. The authors concluded that in utero exposure to opioids has an impact on the neuronal development of the offspring with long-term potentially transmissible repercussions. Additionally, they reported that opioid use before conception also influences the reactivity to opioids of the progeny and the subsequent generations, likely through epigenetic mechanisms.

In conclusion, this research topic provides updated studies and reviews concerning the pharmacology and the toxicology of NSOs as an eye opener of this incoming hazard to scientists and health professionals operating in the field of psychotropic drugs.

## Author Contributions

All authors listed have made a substantial, direct and intellectual contribution to the work, and approved it for publication.

### Conflict of Interest Statement

The authors declare that the research was conducted in the absence of any commercial or financial relationships that could be construed as a potential conflict of interest.

## References

[B1] Busardò F. P. Carlier J. Giorgetti R. Tagliabracci A. Pacifici R. Gottardi M.. (2019). Ultra-high-performance liquid chromatography-tandem mass spectrometry assay for quantifying fentanyl and 22 analogs and metabolites in whole blood, urine, and hair. Front. Chem. 7:184. 10.3389/fchem.2019.00184 31001514 PMC6454115

[B2] Marchei E. Pacifici R. Mannocchi G. Marinelli E. Busardò F. P. Pichini S. (2018). New synthetic opioids in biological and non-biological matrices: a review of current analytical methods. Trends Anal. Chem. 102, 1–15. 10.1016/j.trac.2018.01.007

[B3] Pichini S. Solimini R. Berretta P. Pacifici R. Busardò F. P. (2018). Acute intoxications and fatalities from illicit fentanyl and analogues: an update. Ther. Drug Monit. 40, 38–51, 10.1097/FTD.0000000000000465 29120973

